# A Secure and Efficient Audit Mechanism for Dynamic Shared Data in Cloud Storage

**DOI:** 10.1155/2014/820391

**Published:** 2014-05-12

**Authors:** Ohmin Kwon, Dongyoung Koo, Yongjoo Shin, Hyunsoo Yoon

**Affiliations:** Department of Computer Science, Korea Advanced Institute of Science and Technology (KAIST), Daejeon 305-701, Republic of Korea

## Abstract

With popularization of cloud services, multiple users easily share and update their data through cloud storage. For data integrity and consistency in the cloud storage, the audit mechanisms were proposed. However, existing approaches have some security vulnerabilities and require a lot of computational overheads. This paper proposes a secure and efficient audit mechanism for dynamic shared data in cloud storage. The proposed scheme prevents a malicious cloud service provider from deceiving an auditor. Moreover, it devises a new index table management method and reduces the auditing cost by employing less complex operations. We prove the resistance against some attacks and show less computation cost and shorter time for auditing when compared with conventional approaches. The results present that the proposed scheme is secure and efficient for cloud storage services managing dynamic shared data.

## 1. Introduction

Cloud computing is a promising paradigm to create various computing environments such as [1–3]. Cloud service provider (CSP) allows network-connected users to make use of computing resources in a remote location. As the usage of cloud service matures, users try to share their data in cloud storage and process the data efficiently at a low cost [3–5]. Although several CSPs such as Google [6] and Amazon [7] support computing environments for shared data, integrity of outsourced data is hard to be guaranteed. Due to the lack of transparency, users delegate the control for data management to the third-party CSP but there is no way for users to be noticed about data loss or modification occurred at the cloud storage. In addition, for the reputation of the cloud service, CSPs are reluctant to reveal data inconsistency caused by external threats, software/hardware failures, inside attacks, and so on. Therefore, audit mechanisms are required for verifying consistent data management in the cloud storage.

There are several studies verifying integrity of outsourced data at untrusted storages [8–18]. Most of them [8–14] are yet to consider a situation where the same data is shared by multiple users. In these approaches, only a single user is allowed to update his own data. And he can audit the data either by himself [8, 9, 13] or with assistance from a third-party auditor (TPA) [10–12, 14]. Recent studies [16–18] consider audit for shared data but they only support a limited number of data updates. In addition, the CSP can cheat on censorship in these schemes since an index table used for verification is managed only by the CSP. One way to prevent such a cheat is to make users and the TPA also maintain the index table. Owing to storage and synchronization overhead, however, it might cause a significant delay and degrade the quality of service (QoS) as the number of data updates increases.

In order to design a secure and efficient audit mechanism for dynamic shared data in cloud storage, aforementioned challenges should be efficiently addressed. In other words, the scheme must guarantee the following properties.

1. *Audit for Outsourced Data*. The TPA is able to check the integrity of outsourced data without retrieving all data contents.
2. *Shared Dynamic Data*. Users are allowed to outsource, share, insert, delete, or modify their data contents without restriction.
3. *Efficiency*. Computational overhead for data outsourcing and update at users side as well as the ones for auditing at the TPA should be low.
4. *Soundness*. The CSP is not allowed to deceive users or the TPA into passing a censorship of damaged data contents.

We propose an audit mechanism satisfying the above requirements by utilizing aggregate signature [19] and sample auditing [8]. For data integrity and consistency, the TPA manages an index table and the CSP keeps renewing an identifier for data update. In addition, the audit mechanism provides efficiency to users and the TPA through making the auditing operations simple. Specially, in this paper, we consider forge attack and replace attack as regards soundness for the sake of secure audit. These attacks are described in [20] and they can be summarized as follows.* Forge attack* is an attack to forge a verifying term for a data content, which was not actually outsourced by users.* Replace attack* is an attack to pass a censorship by choosing another data content for verification in place of the damaged data content.

The rest of this paper is organized as follows. In Section 2, we introduce related works about auditing data in the cloud storage. In Section 3, issues about index table management are described depending on which entity manages it. In Section 4, we present methods for our audit mechanism. In Section 5, preliminaries used in our work are briefly introduced. In Section 6, a secure and efficient audit mechanism for dynamic shared data is presented. In Section 7, security of the proposed scheme is analyzed. In Section 8, performance evaluations and experimental results show efficiency of our mechanism. Finally, we conclude our work in Section 9.

## 2. Related Work

Ateniese et al. firstly introduced the notion of provable data possession (PDP) in [8] for integrity check of outsourced data in untrusted storage. They could achieve efficient audit with high probability of detection by sampling random blocks from outsourced data instead of downloading the entire data. Since the original PDP does not consider dynamic data, a user should download the whole data and regenerate metadata for verification whenever there is a modification of the outsourced data. To provide audit for dynamic data without retrieving entire data, subsequent works adopted authenticated data structures such as skip list, Merkle tree, or index tables [9, 12, 14–18]. Erway et al. [9] proposed dynamic provable data possession (DPDP) based on rank-based authenticated skip list and Wang et al. [12] presented a mechanism by exploiting Merkle tree. Both schemes require reconstruction of the authenticated data structure when the corresponding data is updated. Another data structure called index table was introduced to handle data updates more efficiently by keeping unique identifier for each data block [14–18].

It is also notable that outsourced data should be audited periodically for verifying consistent data management. Dedicated to this purpose, TPAs can be delegated by users for auditing outsourced data in privacy-preserving manner. Privacy preservation means that the TPA cannot learn any information about the data during audit process. It was achieved through the methods of random masking [11] and bilinear map [14].

## 3. Index Table Management

Previous works [14–18] utilized an index table for efficient data updates. It is composed of indices which represent the sequences of data blocks and identifiers. The identifier, which is used in tag (tag is a verifying term, stored with data in the cloud storage, and has consistency with a data block) generation and verification, is a number identifying each data block. It should be defined in order to keep the initial value in all circumstances. Otherwise, a user repeats following operations whenever identifiers of data blocks in the cloud storage are changed. The user downloads data blocks which have changed identifiers, regenerates tags of them, and uploads the tags to the cloud storage.

In this section, we look into security issues and update flows depending on which entity manages an index table. At the security aspect, we check the possibility of forge attack and replace attack. Then, we describe communication flows for getting an identifier of new data block when a user tries to update.

### 3.1. Management by the CSP

**Figure 1:**
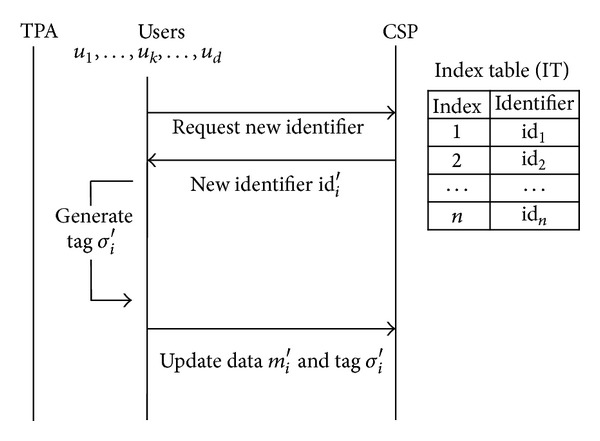
Data update flows when the only CSP manages an index table.

When the CSP only manages an index table, users and the TPA do not have any information about identifiers of data blocks which are already uploaded and will be updated. As illustrated in Figure 1, the user requests an identifier of new data block to the CSP when he tries to update. In this environment, the CSP can forge tags of data blocks which users have not uploaded. It transmits an identifier already existed in the index table then obtains tags which are of different data blocks but have same identifier. It can learn meaningful information for forgery through combination of these data blocks and tags.

Since the TPA needs identifiers of challenged data blocks when verifying, it receives a proof that includes the identifiers from the CSP [16–18]. However, the TPA cannot distinguish them from the challenged ones because it does not maintain an index table. Therefore, if challenged data blocks are modified or deleted, the CSP can replace them with other undamaged data blocks.

### 3.2. Management by the TPA

One simple way to prevent the forge attack and replace attack in the above case is that the TPA manages an index table [15]. Even though the CSP tries to launch a forge attack by exploiting a collision of identifiers, it is impossible because the CSP has no knowledge of identifiers. In addition, a replace attack can be detected easily because the TPA knows identifiers of challenged data blocks through the index table.

**Figure 2:**
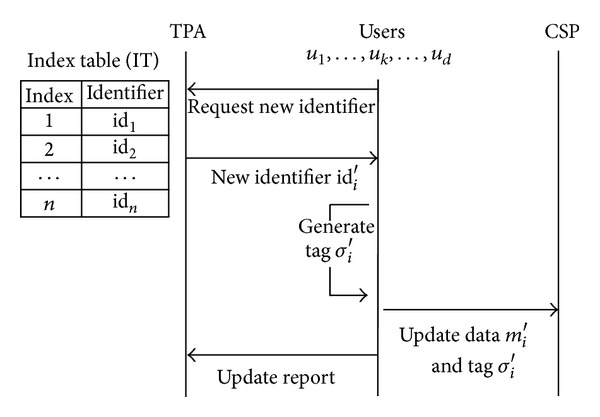
Data update flows when the only TPA manages an index table.

Figure 2 shows that the TPA participates in the update process and a user who tries to update cannot generate a tag for new data block without a reception of new identifier from the TPA. Accordingly, update process can be delayed when the TPA is on sleep or suffers from bottleneck caused by a large number of requests from users.

### 3.3. Management by Both the TPA and Users

Delays of update process can be removed through managing an index table by a user directly [14]. Generally, it is suitable for a situation where data is not shared. However, it has a problem about synchronization of index tables because the index tables are managed separately by each individual user who shares the data. If they are not synchronized, identifiers generated by other users can have same value, then the CSP can exploit forge attack or replace attack by using such tags generated by the same identifiers.

**Figure 3:**
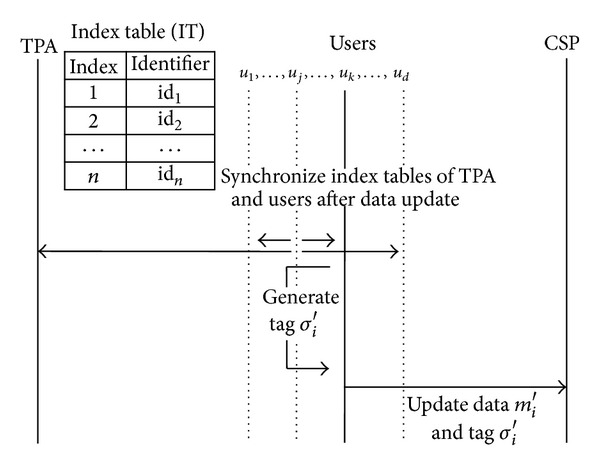
Data update flows when the TPA and users manage an index table together.

Broadcasting update information, after a user finished data update, is a solution for synchronization as shown in Figure 3. However, it requires all users to always wake up. Otherwise, the users need to request the information for synchronization but cannot easily determine who has the newest index table. Although the users can request it to the TPA, this is not different from a previous way that the only TPA manages an index table.

## 4. Methods

In this section, we present methods for a secure and efficient audit mechanism for shared dynamic data.

### 4.1. System Model

**Figure 4:**
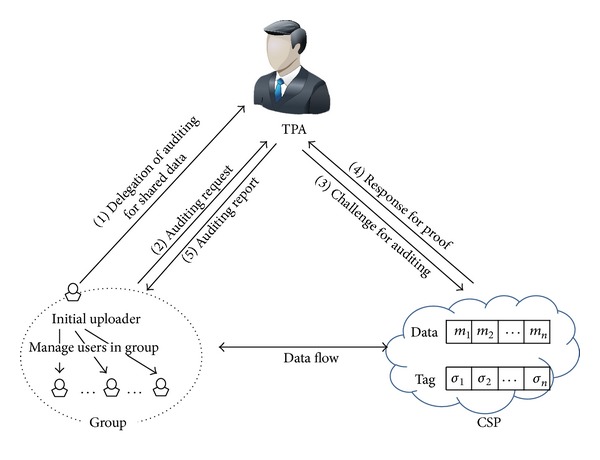
Our system model for auditing mechanism.

Our system model for auditing mechanism is illustrated in Figure 4. There are four entities: CSP, initial uploader, users, and TPA. The CSP provides a large-scale storage for shared data to users. It should process requests from authorized users and respond to every challenge from the TPA. An initial uploader uploads data to cloud storage firstly and forms a group of users who share the data together. Users in the group are able to access and update shared data in the cloud storage. Both an initial uploader and the users are able to audit shared data in cloud storage via the TPA delegated by the initial uploader.

### 4.2. Secure and Efficient Index Table Management

**Figure 5:**
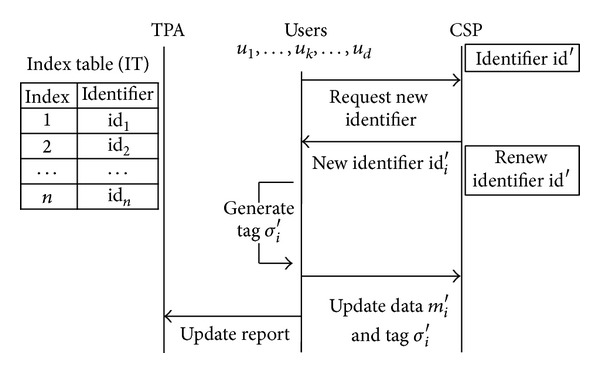
Data update flows in the proposed mechanism.

We propose a secure and efficient index table management that is used for our audit mechanism. As mentioned in the previous section, a TPA must manage an index table to prevent forge attack and replace attack. However, delay and synchronization problems can be caused when the index table is managed by the only TPA or each user. To solve these problems, it is required that a user who tries to update obtains an identifier from the CSP, as described in Figure 5. Consequently, a way that the TPA manages an index table and the CSP keeps renewing an identifier for new data block satisfies security and efficiency.

### 4.3. Identifier Definition for Dynamic Data

Changing identifiers of data blocks by update process causes repetitive tasks to users. They download the corresponding data blocks, regenerate tags to apply the modified identifiers, and upload the tags again to cloud storage. Our mechanism removes these repetitive tasks by defining the identifier as an upload sequence of the data block. If an update of data block happens, then new identifier is assigned as the upload sequence from the CSP.

### 4.4. Simple Operations for Audit Mechanism

Conventional approaches employ relatively complex operations for audit mechanism. It may cause more delay time and computational overhead for tag generation and verification. For efficiency of audit mechanism, the proposed scheme utilizes simple operations in the data integrity check. Moreover, because it can reduce delay time and computational overhead through light-weight operations, the QoS of the cloud storage service is improved.

## 5. Preliminaries

In this section, cryptographic backgrounds for the proposed scheme are briefly introduced.

### 5.1. Bilinear Map

Let *G*_1_ and *G*_2_ be multiplicative cyclic groups of prime order *p*, and let *g* be a generator of *G*_1_. Then a bilinear map *e* satisfies the following properties.

1. *Bilinearity*: for any *u*, *v* ∈ *G*_1_, and *a*, *b* ∈ *Z*_*p*_,  *e*(*u* ^*a*^, *v* ^*b*^) = *e*(*u*, *v*)^*ab*^.
2. *Nondegeneracy*: *e*(*g*, *g*) ≠ 1.
3. *Computability*: there exists an efficiently computable algorithm *e* satisfying the above properties such that *e* : *G*_1_ × *G*_1_ → *G*_2_.

### 5.2. Pseudorandom Permutation

Let *F* : {0,1}^*n*^ × {0,1}^*n*^ → {0,1}^*n*^ be an efficiently computable keyed permutation for positive integer *n*. We say that *F* is a pseudorandom permutation, if there exists a negligible function negl for any probabilistic polynomial-time distinguisher *D* such that

(1) $$\begin{array}{c} \left|\text{Pr}\left[D^{F_{k}(\cdot )}\left(1^{n}\right)=1\right]-\text{Pr}\left[D^{f(\cdot )}\left(1^{n}\right)=1\right]\right|\leq \text{negl}\left(n\right), \end{array}$$

where $k\overset{\text{\$}}{\leftarrow }{\left\{0,1\right\}}^{n}$ is a permutation key chosen uniformly at random and *f* is a real-random permutation.

### 5.3. Discrete Logarithm (DL) Assumption

Discrete logarithm (DL) problem is to compute *a* ∈ *Z*_*p*_, given *g* ∈ *G*_1_ and *g* ^*a*^ ∈ *G*_1_. The DL assumption holds in *G*_1_ if it is computationally infeasible to solve DL problem in *G*_1_.

## 6. The Proposed Scheme

In this section, we present a secure and efficient audit mechanism supporting dynamic updates of shared data. When cloud storage service initiates, a CSP generates public parameters for system and publicizes them. An initial uploader generates secret components and public components used in tag generation and verification. He divides data into blocks and generates tags for each data block. Then, he uploads them to the cloud storage and deletes them in his local storage. To share the data with other users, he needs to deliver secret components for tag generation. In addition, he can delegate auditing processes to a TPA by delivering a part of secret components for verification.

When a user tries to update a new data block, he receives an identifier from the CSP, generates a tag for the data block, and uploads them. After update is finished, the user reports update information to the TPA. Then the TPA updates an index table following the update information. And the CSP renews the identifier as next upload sequence for next update.

The TPA maintains the newest index table that keeps track of upload sequence of data blocks. The TPA makes a challenge derived from the index table and transmits it to the CSP periodically or when a user wants. The CSP who receives the challenge makes a proof and responds to it. Then, the TPA checks whether outsourced data is damaged or not by verifying the proof with the challenge.

### 6.1. Definition

The proposed scheme, Π, is composed of the following eight algorithms such as Π = {*Setup*, *KeyGen*, *TagGen*, *TagGen* *U* *p*, *ITUp*, *ChalGen*, *ProofGen*, *Verify*}.

*Se* *tu* *p*(*γ*) → *param*. On security parameter *γ*, the CSP generates and publicizes public parameters *param*. 

*Ke* *yG* *en*(*param*)→(*gmsk*, *gsk*, *gak*, *gpk*). An initial uploader takes public parameter *param* as input and outputs group master key *gmsk*, group secret key *gsk*, group auditing key *gak*, and group public key *gpk*. Group master key *gmsk* is kept secret. Group secret key *gsk* = {*gsk*_1_, *gsk*_2_} and group auditing key *gak* are used in tag generation. Group public key *gpk* = {*gpk*_1_, *gpk*_2_} is used for verification of data integrity along with group auditing key *gak*. 

*Ta* *gG* *en*(*M*, *gsk*, *gak*) → *σ*. On uploading data *M* to the cloud storage, the initial uploader divides the data into blocks and generates a set of tags *σ* using *gsk* and *gak*. Each component of *σ* is a tag of the corresponding data block in *M* such that *σ* = {*σ*_1_,…, *σ*_*n*_}.

*Ta* *gG* *en* *Up*(*m*_*i*_′, *gsk*, *gak*, id_*i*_′) → *σ*_*i*_′. When a user tries to update a data block *m*_*i*_′, he generates a new tag *σ*_*i*_′ using *gsk*, *gak*, and new identifier id_*i*_′ received from the CSP. 

*IT* *Up*(*U*_type_, *U*_index_, *U*_id_, iT) → iT′. This algorithm takes update information and current index table iT as input and outputs new index table iT′. The update information includes three elements *U*_type_, *U*_index_, and *U*_id_. *U*_type_ represents an update type which can be either insertion, modification, or deletion. *U*_index_ is the index of the data block to be updated, and *U*_id_ is a newly assigned unique identifier used in *TagGen* *U* *p* for the data block. 

*Ch* *al* *G* *en*(iT) → *chal*. This algorithm generates a challenging query *chal* = {*l*, *r*_*l*_}_*l*∈*L*_ for randomly selected blocks. The TPA chooses random indices *L* from the index table iT and generates random values {*r*_*l*_}_*l*∈*L*_. 

*Pr* *oo* *fG* *en*(*M*, *σ*, *chal*, *gpk*)→(*α*, *β*). The CSP generates a proof (*α*, *β*) for a challenge from the TPA. *α* is derived from outsourced data blocks {*m*_*l*_}_*l*∈*L*_, {*r*_*l*_}_*l*∈*L*_, and *gpk*, while *β* is computed from tags {*σ*_*l*_}_*l*∈*L*_ and {*r*_*l*_}_*l*∈*L*_. 

*Ve* *ri* *fy*(*chal*, iT, (*α*, *β*), *gpk*, *gak*) → *True*/*False*. This algorithm verifies consistency of the proof generated by the CSP for the given challenge. If they are consistent, it outputs *True*. Otherwise, it outputs *False*. 

### 6.2. Construction

Let *G*_1_, *G*_2_ be multiplicative cyclic groups of prime order *p*, let *g* be a generator of *G*_1_, let *e* : *G*_1_ × *G*_1_ → *G*_2_ be a bilinear map, and let *F* : {0,1}^log⁡*p*^ × {0,1}^log⁡*p*^ → {0,1}^log⁡*p*^ be a pseudorandom permutation. We consider data *M* is divided into *n* blocks as *M* = (*m*_1_,…, *m*_*n*_), and each data block *m*_*i*_ = (*m*_*i*1_,…, *m*_*is*_) contains *s* sectors of *Z*_*p*_.

#### 6.2.1. Setup

When cloud storage service initiates, the CSP chooses two groups *G*_1_, *G*_2_ with *g* for a bilinear map *e* by running *Setup* algorithm and publicizes *param* = (*G*_1_, *G*_2_, *p*, *g*, *e*) to users and the TPA.

#### 6.2.2. Upload for Data Sharing

An initial uploader, *u*_1_, runs *KeyGen* algorithm. It first chooses (*s* + 2) random values *gmsk* = *a* ∈ *Z*_p_*, *gak* = *b* ∈ *Z*_*p*_, and *gsk*_2_ = {*gsk*_2,*j*_ = *c*_*j*_ ∈ *Z*_*p*_}_*j*∈[1,*s*]_ and computes *gsk*_1_ = *g* ^1/*a*^,  *gpk*_1_ = *g* ^*a*^, and *gpk*_2_ = {*gpk*_2,*j*_ = *g* ^*c*_*j*_^}_*j*∈[1,*s*]_. Then, by running *TagGen*, *u*_1_ generates corresponding tags such as

(2) $$\begin{array}{c} \sigma_{i}={\left(gsk_{1}\right)}^{F_{gak}(i)\;+\sum_{j\in [1,s]}^{}gsk_{2,j}\cdot m_{ij}} \end{array}$$

for 1 ≤ *i* ≤ *n*. *u*_1_ uploads (*M*, *σ*) to the cloud storage and deletes them from his local storage. When the CSP receives fresh data from the initial uploader, it saves an identifier id′ = *n* + 1 for the next upload sequence. To share data *M* with other users, *u*_1_ delivers group secret key *gsk* and group auditing key *gak* to them.

#### 6.2.3. Delegation of Audit

The initial uploader *u*_1_ delegates audit for shared data to the TPA by delivering a group auditing key *gak*. In addition, *u*_1_ notifies the number of data blocks *n* for the TPA to correctly generate an initial index table for *M*. In other words, the TPA creates an index table which includes identifiers defined as block sequences initially.

#### 6.2.4. Data Update

In the proposed scheme, any user *u*_*k*_ in the group which shares data *M* is allowed to modify, insert, or delete data *M* in the cloud storage.

If *u*_*k*_ tries to modify *i*-th block *m*_*i*_, *u*_*k*_ first receives a new identifier from the CSP as illustrated in Figure 5. On receipt of the identifier id_*i*_′, *u*_*k*_ computes a tag *σ*_*i*_′ for updated data block *m*_*i*_′ by running *TagGen* *U* *p* algorithm as follows:

(3) $$\begin{array}{c} \sigma_{i}^{\prime }={\left(gsk_{1}\right)}^{F_{gak}({\text{id}}_{i}^{\prime })\;+\sum_{j\in [1,s]}^{}gsk_{2,j}\cdot m_{ij}^{\prime }}. \end{array}$$

Then, *u*_*k*_ sends (*U*_type_ = `*M*′, *U*_index_ = *i*, *U*_id_ = id_*i*_′) along with (*m*_*i*_′, *σ*_*i*_′) to the CSP, where `*M*′ stands for modification. The CSP can update the data block in the cloud storage by replacement of (*m*_*i*_, *σ*_*i*_) with (*m*_*i*_′, *σ*_*i*_′) uploaded by *u*_*k*_.

For the case of insertion, *u*_*k*_ makes a tag *σ*_*i*_′ for newly created data block *m*_*i*_′ by running *TagGen* *U* *p* algorithm and sends (*U*_type_ = `*I*′, *U*_index_ = *i*, *U*_id_ = id_*i*_′) along with (*m*_*i*_′, *σ*_*i*_′) to the CSP.

When *u*_*k*_ wants to delete data block in the cloud storage, *u*_*k*_ sends (*U*_type_ = `*D*′, *U*_index_ = *i*, *U*_id_ = 0) to the CSP and allows the CSP to delete the corresponding data block from the cloud storage. In this case, *u*_*k*_ does not need to request a new identifier. Therefore, we set the value of *U*_id_ by zero.

**Figure 6:**
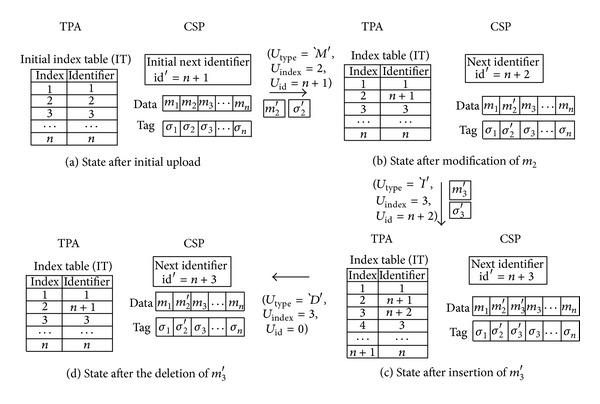
Examples of data update.

After update is finished, *u*_*k*_ delivers update information (*U*_type_, *U*_index_, *U*_id_) to the TPA for consistent audit for updated shared data in the cloud storage. Then, the TPA updates the index table managed by itself according to the information by running *ITUp*. When *U*_type_ = `*M*′, it changes the *U*_index_-th identifier to *U*_id_. When *U*_type_ = `*I*′, the TPA inserts *U*_id_ just before the *U*_index_-th field, while it removes the *U*_index_-th field from the index table if *U*_type_ = `*D*′. Through this notification of data updates, the TPA can detect malicious behaviors of the CSP. In other words, the CSP cannot transmit previously used identifier in order to exploit forge attack and replace attack. Simple examples of data update are depicted in Figure 6.

#### 6.2.5. Audit for Outsourced Data

For the TPA to check integrity of outsourced data, it engages in challenge-response protocol with the CSP. The TPA first chooses a random subset *L* of indices from the index table and generates random values {*r*_*l*_ ∈ *Z*_p_*}_*l*∈*L*_ by running *ChalGen* algorithm. The *chal* = {*l*, *r*_*l*_}_*l*∈*L*_ is transmitted to the CSP and the CSP generates a proof (*α*, *β*) for the challenge *chal* by running *ProofGen* algorithm as follows:

(4) $$\begin{array}{c} \alpha =\underset{j\in [1,s]}{\prod }gpk_{2,j}^{\sum_{l\in L}^{}r_{l}\cdot m_{lj}},\\ \beta =\underset{l\in L}{\prod }\sigma_{l}^{r_{l}}. \end{array}$$

After receiving the proof (*α*, *β*) as a response of the challenge, the TPA verifies it by running *Verify* algorithm as follows:

(5) $$\begin{array}{c} e\left(g^{\sum_{l\in L}^{}r_{l}\cdot F_{gak}({\text{id}}_{l})}\cdot \alpha ,g\right){=}^{?}e\left(\beta ,gpk_{1}\right). \end{array}$$

If (5) holds then the TPA returns *True* and returns *False* otherwise.

## 7. Security Analysis

In this section, we show that the proposed scheme is correct and resistant against forge attack and replace attack.

**Theorem 1**

  Π provides correctness to the TPA during auditing outsourced data.

**Proof**

Correctness of Π is achieved by exploiting bilinear property of the bilinear map. Left-hand side (LHS) of (5) expands as follows:

(6) LHS=e(g∑l∈Lrl·Fgak(idl)·∏j∈[1,s]ggsk2,j·∑l∈Lrl·mlj,g)=e(g∑l∈Lrl·Fgak(idl)+∑j∈[1,s](gsk2,j·∑l∈Lrl·mlj),g)=e(g,g)∑l∈L(rl·Fgak(idl)+rl·∑j∈[1,s]gsk2,j·mlj)

while the right-hand side (RHS) of (5) expands as follows:

(7) RHS=e(∏l∈Lσlrl,gpk1)=e(∏l∈Lgrl·(Fgak(idl)+∑j∈[1,s]gsk2,j·mlj)/gmsk,ggmsk)=e(g∑l∈L(rl·Fgak(idl)+rl·∑j∈[1,s]gsk2,j·mlj)/gmsk,ggmsk)=e(g,g)∑l∈L(rl·Fgak(idl)+rl·∑j∈[1,s]gsk2,j·mlj).

Since the terms LHS and RHS are the same, the proof is completed.

**Theorem 2**

Π is secure against forge attack.

**Proof**

If the CSP acquires an element in {*gpk*_2,*j*_^1/*gmsk*^ = *g* ^*gsk*_2,*j*_/*gmsk*^}_*j*∈[1,*s*]_ from the public parameter *gpk*, the CSP can forge a tag. Given *gpk*_1_ = *g* ^*gmsk*^, computing *gmsk* is hard due to DL assumption. Thus, it is infeasible for the CSP to acquire an element in {*g* ^*gsk*_2,*j*_/*gmsk*^}_*j*∈[1,*s*]_.

When *F*_*gak*_(id_*i*_) for *σ*_*i*_ and *F*_*gak*_(id_*q*_) for *σ*_*q*_ are the same, the CSP computes *σ*_*i*_/*σ*_*q*_. Then, the CSP can obtain *δ* = (*gsk*_1_)^∑_*j*∈[1,*s*]_*gsk*_2,*j*_·(*m*_*ij*_−*m*_*qj*_)^ and forge the tag *σ*_*z*_ of data block *m*_*z*_ using *δ* as follows:

(8) σz′=σz×δ=(gsk1)Fgak(idz)+∑j∈[1,s]gsk2,j·mzj×(gsk1)∑j∈[1,s]gsk2,j·(mij−mqj)=(gsk1)Fgak(idz)+∑j∈[1,s]gsk2,j·(mzj+mij−mqj).

Finally, the CSP obtains forged tag *σ*_*z*_′ of modified data block *m*_*z*_′ = {*m*_*zj*_ + *m*_*ij*_ − *m*_*qj*_}_*j*∈[1,*s*]_. However, *F*_*gak*_(id_*i*_) and *F*_*gak*_(id_*q*_) cannot be the same value for id_*i*_ ≠ id_*q*_ because of the definition of pseudorandom permutation. Therefore, the CSP cannot forge a tag to pass the censorship. This completes the proof.

**Theorem 3**

Π is secure against replace attack.

**Proof**

When damaged block *m*_*i*_ is challenged, the CSP may try to pass the censorship by choosing a different block (*m*_*q*_, *σ*_*q*_) in place of (*m*_*i*_, *σ*_*i*_) such as *α*′ = ∏_*j*∈[1,*s*]_ *gpk*_2,*j*_^*r*_*i*_·*m*_*qj*_+∑_*l*∈*L*,*l*≠*i*_*r*_*l*_·*m*_*lj*_^ and *β*′ = *σ*_*q*_^*r*_*i*_^ · ∏_*l*∈*L*,*l*≠*i*_ *σ*_*l*_^*r*_*l*_^. Then, the left-hand side of (5) is computed as

(9) e(g∑l∈Lrl·Fgak(idl)·∏j∈[1,s]gpk2,jri·mqj+∑l∈L,l≠irl·mlj,g)=e(g∑l∈Lrl·Fgak(idl)·g∑j∈[1,s]gsk2,j·(ri·mqj+∑l∈L,l≠irl·mlj),g)=e(gri·(Fgak(idi)+∑j∈[1,s]gsk2,j·mqj)+∑l∈L,l≠irl·(Fgak(idl)+∑j∈[1,s]gsk2,j·mlj),g)=e(g(ri·Fgak(idi)−ri·Fgak(idq))/gmsk×β′,gpk1)

in which *g* ^(*r*_*i*_·(*F*_*gak*_(id_*i*_)−*F*_*gak*_(id_*q*_)))/*gmsk*^ should be 1 to satisfy (5). This means that *F*_*gak*_(id_*i*_) and *F*_*gak*_(id_*q*_) should be the same. Due to the bijective property of the permutation, however, these values cannot be the same.

## 8. Performance Evaluation

In this section, the prosed scheme is analyzed and compared with previous studies [14–16] in terms of communication and computational overhead. We first evaluate communication overhead for updating a data block. Then, computational overhead for tag generation and verification is evaluated.

### 8.1. Communication Overhead

As we described in Section 3, the way to get an identifier of updated data block depends on which entity manages an index table. In Wang et al.'s work [16], *u*_*k*_ needs one round-trip communication to request and receive the identifier (Figure 1). Zhu et al. [15] utilize a way that the TPA manages the index table (Figure 2). It needs an additional connection between *u*_*k*_ and the TPA for the identifier and a report of update information. Yang and Jia [14] utilize a way that a user manages the index table by himself. Although it is suitable when the outsourced data is managed by a single user, it requires more communication costs for synchronization of the index tables when the data is shared by multiple users.

Communication costs are summarized in Table 1. We omitted costs for uploading a data block and a corresponding tag for simplicity. Although the proposed scheme seems to require the same cost as Zhu et al.'s approach, there may be update delays caused by concentration of communications to the TPA in [15]. On the other hand, the proposed scheme removes this delay via a direct acquisition of the identifier from the CSP. For [14] to synchronize the index tables of users and the TPA, *u*_*k*_ needs to broadcast extra update information. Considering this circumstance, additional communications caused by broadcast might be added into Table 1.

### 8.2. Computational Overhead

Computation costs for tag generation and verification are described in Table 2. The proposed scheme requires a single Exp_*G*_ operation in tag generation, while the others [14–16] require Exp_*G*_ and Mul_*G*_ operations which are proportional to the number of sectors in a data block. When the TPA verifies a proof received from the CSP, one Exp_*G*_ and one Mul_*G*_ operations are required in the proposed scheme regardless of the number of challenged data blocks. However, the others require Exp_*G*_ and Mul_*G*_ operations linear to the number of challenged data blocks, which cause a significant overhead to the TPA.

### 8.3. Experimental Results

We measure the performance of our scheme and compare it with other works [14–16] based on implementations in Ubuntu 12.04. We utilize Paring Based Cryptography (PBC) library for cryptographic operations and OpenSSL to use Advanced Encryption Standard (AES) for pseudorandom permutation. All experiments are executed on an Intel Core i3 3.10 GHz with 2 GB memory. We assume that |*p*| is 160 bits, |id| is 80 bits, and size of a data block is 160 bits. We simulate each scheme on 4 different data which has 1,000 data blocks with 5 times. All experimental results show an average of 20 trials.

#### 8.3.1. Tag Generation

**Figure 7:**
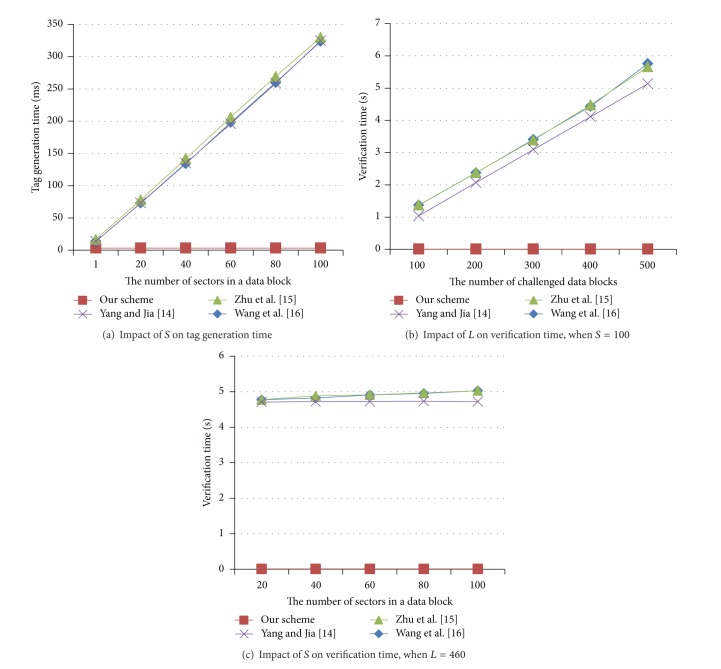
Experimental results.

The performances of the tag generation times are presented in Figure 7(a). Tag generation time in our scheme is 3.18 milliseconds per block when *S* = 1 and 3.28 milliseconds per block when *S* = 100. Since a single Exp_*G*_ is required regardless of *S*, *S* almost never influences on tag generation time. However, tag generation times of the others increase with increasing *S*. Reference [16] requires 323.65 milliseconds per block, and [14] requires 324.63 milliseconds per block when *S* = 100. Since they have same computation complexity, their tag generation times are almost identical. Reference [15] needs one more Exp_*G*_. Thus, it requires 329.97 milliseconds per block which is more than the others.

#### 8.3.2. Verification

We measure verification times depending on *L* when *S* = 100. As depicted in Figure 7(b), the verification times for the TPA are dependent on *L* in the others [14–16]. When *L* = 500, [14–16] requires 5.13, 5.66, and 5.75 seconds, respectively. Furthermore, Figure 7(c) shows that *S* influences [15, 16]. However, our scheme requires 0.01 seconds for verification regardless of *L* and *S*.

## 9. Conclusion

In this paper, we present a secure and efficient audit mechanism for shared dynamic data in cloud storage. It makes possible for the TPA to correctly audit outsourced data which can be updated in a secure manner. With simple index table management by the TPA and identifier renewal by the CSP, any user in a group can update shared data block efficiently. Furthermore, making the auditing operations simple leads to less computational overhead for the whole auditing process. Performance evaluation and security analysis show that the proposed scheme is best suited to the cloud storage where multiple users share and update the outsourced data frequently.

## Figures and Tables

**Table 1:** Connections and communication costs for updating a data block.

	Wang et al. [16]	Zhu et al. [15]	Yang and Jia [14]	Our scheme
Connections	*u*_*k*_↔CSP	*u*_*k*_↔TPA	*u*_*k*_ with other users	*u*_*k*_↔TPA
		*u*_*k*_↔CSP	*u*_*k*_↔TPA	*u*_*k*_↔CSP
			*u*_*k*_↔CSP	

Extra communication costs	|*q*_id_| + |id|	|*q*_id_| + |id| + |*u*_info_|	*d* × |*u*_info_|	|*q*_id_| + |id| + |*u*_info_|

**Table 2:** Computation costs for a tag generation and verification.

	Tag generation	Verification
Wang et al. [16]	(*S* + 1) · Exp_*G*_ + *S* · Mul_*G*_ + *H*_*G*_	2 · Pair + (*L* + *S*) · Exp_*G*_ + (*L* + *S* − 1) · Mul_*G*_ + *L* · *H*_*G*_
Zhu et al. [15]	(*S* + 2) · Exp_*G*_ + *S* · Mul_*G*_ + *H*_*G*_	3 · Pair + (*L* + *S*) · Exp_*G*_ + (*L* + *S*) · Mul_*G*_ + *L* · *H*_*G*_
Yang and Jia [14]	(*S* + 1) · Exp_*G*_ + *S* · Mul_*G*_ + *H*_*G*_	2 · Pair + (*L* + 1) · Exp_*G*_ + *L* · Mul_*G*_ + *L* · Mul_*Z*_*p*__ + *L* · *H*_*G*_
Our scheme	Exp_*G*_ + *S* · Mul_*Z*_*p*__ + *S* · Add_*Z*_*p*__ + *F*_prp_	2 · Pair + Exp_*G*_ + Mul_*G*_
		*L* · Mul_*Z*_*p*__ + (*L* − 1) · Add_*Z*_*p*__ + *L* · *F*_prp_

## Notations

*u*_*k*_: A user who tries to update
*d*: The number of users in group
*L*: The number of challenged data blocks
*S*: The number of sectors in a data block
id: An identifier
*q*_id_: Request for an identifier
*u*_info_: Update information
*F*_prp_: Pseudorandom permutation
*H*_*G*_: {0,1}* to *G*
Pair: Pairing operation
Exp_*G*_: Exponentiation in *G*
Mul_*G*_: Multiplication in *G*
Mul_*Z*_*p*__: Multiplication in *Z*_*p*_
Add_*Z*_*p*__: Addition in *Z*_*p*_.
